# A simplified similarity-based approach for drug-drug interaction prediction

**DOI:** 10.1371/journal.pone.0293629

**Published:** 2023-11-09

**Authors:** Guy Shtar, Adir Solomon, Eyal Mazuz, Lior Rokach, Bracha Shapira

**Affiliations:** 1 Department of Software and Information Systems Engineering, Ben-Gurion University of the Negev, Beer-Sheva, Israel; 2 Department of Information Systems, University of Haifa, Haifa, Israel; Università degli Studi di Milano, ITALY

## Abstract

Drug-drug interactions (DDIs) are a critical component of drug safety surveillance. Laboratory studies aimed at detecting DDIs are typically difficult, expensive, and time-consuming; therefore, developing in-silico methods is critical. Machine learning-based approaches for DDI prediction have been developed; however, in many cases, their ability to achieve high accuracy relies on data only available towards the end of the molecule lifecycle. Here, we propose a simple yet effective similarity-based method for preclinical DDI prediction where only the chemical structure is available. We test the model on new, unseen drugs. To focus on the preclinical problem setting, we conducted a retrospective analysis and tested the models on drugs that were added to a later version of the DrugBank database. We extend an existing method, adjacency matrix factorization with propagation (AMFP), to support unseen molecules by applying a new lookup mechanism to the drugs’ chemical structure, lookup adjacency matrix factorization with propagation (LAMFP). We show that using an ensemble of different similarity measures improves the results. We also demonstrate that Chemprop, a message-passing neural network, can be used for DDI prediction. In computational experiments, LAMFP results in high accuracy, with an area under the receiver operating characteristic curve of 0.82 for interactions involving a new drug and an existing drug and for interactions involving only existing drugs. Moreover, LAMFP outperforms state-of-the-art, complex graph neural network DDI prediction methods.

## 1 Introduction

Adverse drug reactions are estimated to be the fourth major source of mortality in the United States, before pulmonary illness, diabetes, AIDS, pneumonia, accidents, and vehicular deaths [1]. The number of patients injured by drug interactions is estimated to represent 3-5 percent of all patients harmed by medication mistakes. Drug interactions also account for many patient visits to doctors and emergency rooms [2, 3]. It is challenging to detect drug-drug interactions (DDIs) during the clinical trials before a drug is approved [4]. As a result, potential DDIs are generally not detected until the third phase of a clinical study or after the treatment is already on the market. A drug can potentially interact with any of the few thousand approved drugs. Given the vast number of drug combinations, in-silico drug-drug interaction detection is the most practical technique for screening interacting medications.

In recent years, researchers have gathered drug data from the literature, reports, and other sources to create databases that can aid in developing in-silico DDI prediction methods. As a result, machine learning approaches for DDI prediction have gained popularity, saving time and money [5]. These methods can be categorized into two groups: (1) preclinical DDI prediction: methods which use the chemical structure of a drug as input [6–8]; and (2) modality-intensive DDI prediction: methods that use a single domain-expert-engineered drug feature (or fuse several of these features), such as the known drug-drug interactions, drug-target interactions, and side effects [9–12] of a given drug, to predict its DDIs. The main limitation of modality-intensive DDI prediction methods stems from the fact that the required domain-expert-engineered drug features are not available until the advanced stages of the drug lifecycle [13]. Thus, the models’ predictions based on these features are only available after the drug has been clinically tested or even approved. Furthermore, modality-intensive DDI prediction requires significant human resources, time, and effort. Therefore, in this study, we focus on the preclinical DDI prediction task, a task which is quite challenging due to the lack of handcrafted features.

Recent works demonstrated that known drug-drug interactions are very accurate predictors of new interactions [11], and outperform other modality-intensive methods which incorporate many drug features. Using known DDIs to predict unknown ones, the problem can be tackled as a classical link prediction problem and solved using matrix factorization (MF) techniques, a way to break down large matrices into simpler, more manageable forms. This solution is analogous to collaborative filtering in recommender systems, a technique where recommendations are made based on the preferences of similar users. Collaborative filtering is usually performed using MF techniques, which generally perform better than methods that use content-based information or meta-data regarding the items and users [14]. This is somewhat like recommending movies to a friend based on what their similar friends like. Recently, these MF techniques have gained popularity in drug modelling [15, 16].

Motivated by methods that employ MF techniques, we use MF as part of our DDI prediction method in this study. From a recommender systems perspective, the preclinical DDI prediction task is similar to the *cold-start* task, a situation where we have little to no previous data. In preclinical DDI prediction, we are facing the same challenges created by insufficient data. Our approach for predicting preclinical DDIs is inspired by certain techniques in recommendation systems. Specifically, we looked at solutions for the cold-start problem [17, 18]. Here, we extend the architecture of adjacency matrix factorization with propagation (AMFP), which uses known DDIs to predict new ones. We introduce the lookup adjacency matrix factorization with propagation (LAMFP) which performs matrix factorization on the adjacency matrix and propagates each drug’s representation to interacting drugs. In simpler terms, LAMFP looks at the relationships between drugs and uses this to make educated guesses about new, unseen drugs. LAMFP deals with unseen drugs by employing a simple similarity-based mechanism, the lookup mechanism, to replace unseen drugs with known drugs. We show that our method outperforms state-of-the-art solutions and complex deep learning architectures like directed message passing neural networks [19] for the preclinical drug-drug interaction prediction task.

In this study, we leverage the molecular structure, which is available at any stage of the drug development process. We predict DDIs by looking at how similar drugs have interacted in the past. Existing preclinical DDI methods were reported to perform well on a holdout evaluation scheme with an area under the receiver operating characteristic (AUC) ≥ 0.9 [6, 7], however, they struggle when faced with unseen drugs. As our evaluation demonstrates, methods that model the molecular structure as a graph of atoms and bonds or process the simplified molecular-input line-entry system (SMILES) representation using neural networks underperform compared to the proposed straightforward, molecular similarity-based method when evaluated on drug interactions involving a new, unseen drug. Throughout this paper, we consistently refer to “drug interactions” as potential adverse or harmful reactions between drugs. Our primary objective is to identify unknown drug interactions that, as of the current state of knowledge, are believed to be non-existent or unreported.

This study’s main contributions are as follows:

1. We present the LAMFP algorithm, which extends an existing drug-drug interaction method (AMFP) to support unseen drugs by performing a lookup on existing drugs based on their chemical structure.
2. We assess the performance of various chemical structure similarity measures for the task of DDI prediction and propose an ensemble based on several similarity measures.
3. We propose and evaluate several preclinical DDI prediction methods based on recurrent neural networks and message passing neural networks.

The methods are evaluated using retrospective analysis, focusing on new, unseen drugs. The proposed method is compared to existing state-of-the-art methods.

## 2 Methods

In Section 2.1, we outline the drug-drug interaction prediction task. The AMFP prediction method is detailed in Section 2.2, and its extension, LAMFP, which addresses previously unseen drugs, is discussed in Section 2.3.

### 2.1 Problem formulation

We model the preclinical drug-drug interaction prediction as a binary classification problem using the drugs’ chemical structures. The interactions are binary, indicating either presence (1) or absence (0) of interaction based on the chemical structures. Given an existing drug *i*, we use its chemical structure *s*(*i*). Similarly, we use the chemical structure for new drugs $\hat{j}$ and $\hat{l}$ represented by $s(\hat{j})$ and $s(\hat{l})$ respectively. Our goal is to predict whether an interaction will exist between (1) drugs *i* and $\hat{j}$ based on their chemical structure denoted by $interaction(s\left(i\right),s\left(\hat{j}\right))$, and (2) new drugs $\hat{j}$ and $\hat{l}$ based on their chemical structure denoted by $interaction(s\left(\hat{j}\right),s\left(\hat{l}\right))$. The interaction prediction could be one of the following two types of interactions: (1) an interaction that exists, where *interaction*(*s*(⋅), *s*(⋅)) = 1, or (2) an interaction does not exist, where *interaction*(*s*(⋅), *s*(⋅)) = 0.

### 2.2 AMFP

Adjacency matrix factorization (AMF) and adjacency matrix factorization with propagation (AMFP) were developed for DDI prediction by [11]; both methods are based on factorization of the interaction graph adjacency matrix. Techniques based on matrix factorization are widely used in recommender systems [20], where each user and item (i.e. movie) are represented by a compressed latent vector (embedding) that represents the user’s taste. AMF captures each drug’s essence with an embedding, recreating the interaction network from these embeddings.

To calculate the drug’s embedding, we first represent all drug interactions with an adjacency matrix, akin to a friend list in a social network marking who is acquainted with whom. This matrix contains the interactions between all drugs. Following this, we utilize an inner product calculation between all drug vectors. For each row *i* and each column *j*:

$$\begin{array}{c} {\hat{y}}_{i,j}=\sum_{w=1}^{k}p_{iw}q_{jw} \end{array}$$ (1)

In this context, the embeddings, reminiscent of ‘reputation scores’ in our social network analogy, for drugs *i* and *j* are situated in a shared vector space (shared weights), denoted by *p*_*i*_ and *q*_*j*_. The dimension of these embeddings is defined by the parameter *k*. ${\hat{y}}_{i,j}$ acts as an estimator to predict if two drugs, similar to two well-known individuals in our metaphor, might have an interaction. Recognizing the potential pitfalls of an extremely low or high *k*, we further refine our computation:

$$\begin{array}{c} {\hat{y}}_{i,j}=\mu +b_{i}+b_{j}+\sum_{w=1}^{k}p_{iw}q_{jw} \end{array}$$ (2)

Here, *b*_*i*_ and *b*_*j*_ represent bias values for drugs *i* and *j*, analogous to adjusting our perceptions based on someone’s affiliations in the social scenario. The *μ* value is determined from the average value of the entire ‘friend list’. We then optimize these parameters using techniques comparable to fine-tuning our comprehension of a social network. In the final step, this matrix factorization is integrated within a neural network structure.

Despite AMF’s high accuracy in hold-out evaluations, its performance drops in retrospective evaluations. Hence, AMFP introduces “latent factor propagation” to improve generalizability, assuming that interacting drugs share characteristics. However, AMFP struggles with the cold-start problem when it comes to new drugs. AMFP utilizes the same model as AMF, but with an additional step called “latent factor propagation.” This step involves sharing latent factors of each drug with its interacting drugs, controlled by a propagation factor (*α*). The propagation factor determines how much influence the latent factors of neighboring drugs have compared to the original factors optimized in the previous step.

The full algorithm can be found under algorithm one of the original paper [11], here’s a simpler description of the algorithm: for each node in the graph, its latent factor is shared with its neighbors. The propagation factor (*α*) determines the extent of information exchange between the node and its neighbors. Optimizing the value of *α* during training is essential.

In this context, a node represents a drug, and its neighborhood is consists of the interacting drugs. When the propagation factor (*α*) is set to one, the original latent factors are discarded, and new factors are created based on the neighborhood. Conversely, when *α* is zero, the previous step’s latent factors are retained, and the model remains unchanged. If *α* is equal to zero, AMFP and AMF produce equivalent results. The purpose of propagating factors is to enhance the model’s generalization ability by combining factors from interacting drugs, based on the assumption that interacting drugs share common characteristics.

AMF and AMFP were both designed to learn a drug representation from existing interactions, however, this assumption makes these algorithms unsuitable for new drugs where no existing interactions are known; in other words, the algorithm is highly vulnerable to the cold-start problem. In general, as our evaluation shows, it is possible to use the average latent vector, or even a random one, to represent new drugs. Still, the predictions of such a technique would be of limited clinical significance.

Due to AMFP’s strong performance, its simplicity in using a simple, single input of existing DDIs and its ability to support small molecules and biologics with a single model, we focus on extending it to tackle its main drawback—its inability to support unseen drugs.

**Algorithm 1**: LAMFP—Lookup Mechanism

**Input**: new drug *a*, drug *b*, drug similarity measure *F*, threshold of maximum number of similar drugs *m*, set of existing drugs with known interactions *K*

**Output**: a prediction for the existence of a DDI between drugs *a* and *b*.

1: *S*_*a*_ ← *NN*(a, K, m, F)

2: **if** *a* ∉ *K* and *b* ∈ *K* **then**

3:  **for** *c*, *sim* ∈ *S*_*a*_ **do**

4:   *p* ← *p* + *AMFP*(*c*, *b*) × *sim*

5:   *sum* ← *sum* + *sim*

6:  **end for**

7: **else**

8:  **if** *a* ∉ *K* and *b* ∉ *K* **then**

9:   *S*_*a*_ ← NN(*a*,*K*,*m*,*F*)

10:   *S*_*b*_ ← NN(*b*,*K*,*m*,*F*)

11:   **for** *index* = 0, ‥, *m* **do**

12:    *s*_*a*_, *sim*_*a*_ ← *S*_*a*_[*index*]

13:    *s*_*b*_, *sim*_*b*_ ← *S*_*b*_[*index*]

14:    $w\leftarrow \frac{2}{\frac{1}{score_{a}}+\frac{1}{score_{b}}}$

15:    *p* ← *p* + *AMFP*(*s*_*a*_, *s*_*b*_) × *w*

16:    *sum* ← *sum* + *w*

17:   **end for**

18:  **end if**

19: **end if**

20: Return $\leftarrow \frac{p}{sum}$

21:

22: NN(*a*,*K*,*m*,*F*): returns an ordered list of *m* tuples; each tuple consists of an existing drug *c* ∈ *K* and a similarity score *F*(*a*, *c*)

### 2.3 LAMFP

**Fig 1:**
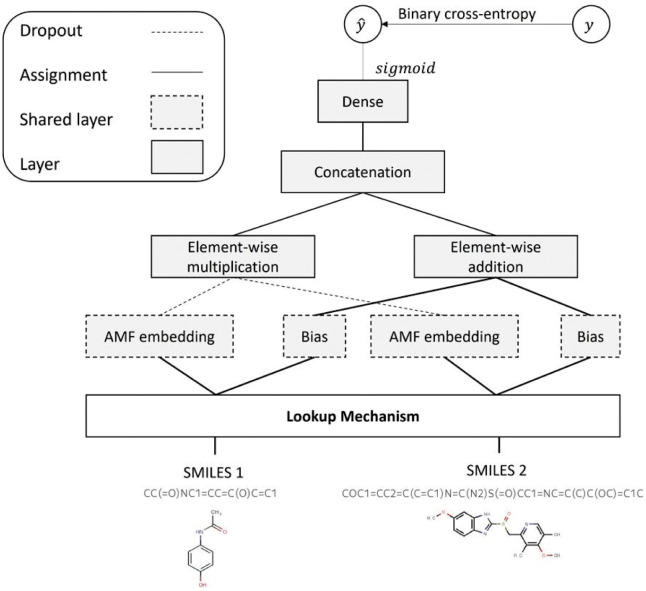
Overview of the LAMFP algorithm’s architecture. Given an unseen drug, a lookup mechanism is used to identify chemically similar drugs which are used as input to AMFP. AMFP performs matrix factorization on the interaction graph adjacency matrix, followed by propagation of the drug’s representation to interacting drugs.

**Fig 2:**
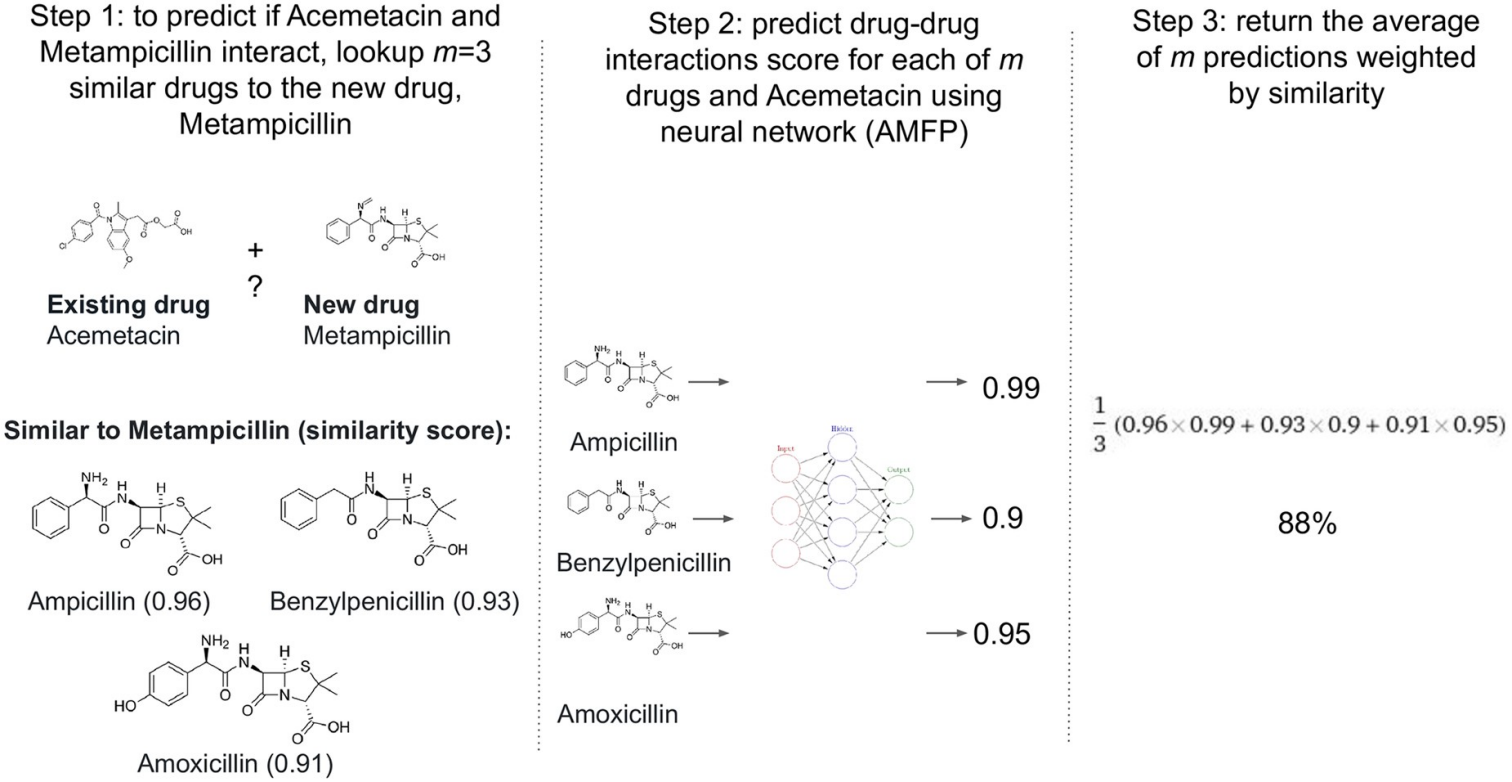
Predicting the existence or absence of interaction between a new and an existing molecule using LAMFP. Step1: a new molecule is lookup using the lookup mechanism, *m* = 3 similar drugs, and the drug’s similarity are retrieved. Step 2: the DDI between the *m* drugs and the existing drug is predict. Step 3: the result is the average score weighted by similarity.

To remedy AMFP’s inability to predict for unseen drugs, we introduce LAMFP. This model employs a *lookup mechanism*, utilizing similarity measures, to base predictions of unseen drugs on the predictions of known drugs. LAMFP deals with two scenarios: predictions involving one unseen drug and predictions involving two unseen drugs. In both cases, the prediction leverages the similarity of the drugs to known drugs and the interactions of those known drugs. For cases where both drugs are known, LAMFP falls back to AMFP. We present an overview of the LAMFP architecture in Fig 1. The LAMFP method uses a *lookup mechanism* in which the prediction of unseen drugs is calculated using the predictions of *m* known drugs; the predictions are based on a similarity measure denoted by *F*. LAFMP’s lookup mechanism is described in detail in Algorithm 1 and visualized in Fig 2.

We define two cases for LAMFP: (1) predicting drug interaction involving a single new, unseen molecule:

$interaction(s\left(i\right),s\left(\hat{j}\right))$ , and (2) predicting drug interaction involving two new molecules which: $interaction(s\left(\hat{j}\right),s\left(\hat{l}\right))$; in the case of the former, we calculate the new drug’s prediction by finding the weighted average of the predictions of the *m* most similar known drugs’ based on *F*. In the latter, the prediction is calculated for two unseen drugs. Therefore, based on *F*, we retrieve the most similar *m* known drugs for $s(\hat{j})$ and $s(\hat{l}))$. Then, we calculate the weighted mean prediction between every two corresponding drugs in the two lists. Weights are found by calculating the harmonic mean of the similarity scores.

In cases where both molecules are known, the LAMFP algorithm uses the AMFP algorithm (i.e., without the lookup mechanism), as described in Section 2.2, to provide DDI predictions.

The lookup mechanism relies on the test-time augmentation (TTA) technique, which was shown to be beneficial for creating robust and accurate machine learning models [21, 22]. The use of this mechanism enables AMFP to handle unseen drugs and provide more accurate DDI predictions.

#### Similarity Measures

LAMFP accommodates different similarity measures. We experimented with:

- Tanimoto Similarity—Measures the shared chemical substructures. It is given by calculating the 2048-bit Morgan fingerprints and determining the proportion of shared chemical substructures using Tanimoto similarity, as described by [23].

These measures represent a type of similarity measure based directly on the molecules’ chemical structure. The second type of similarity measure that we examine is based on the SMILES representation derived from the chemical structure. The following SMILES-based similarity measures are tested:

- Edit distance (ED) [24, 25]—Computes the similarity based on the minimum edit operations (insertion, deletion, substitution) needed to convert one SMILES representation to another:
  $$\begin{array}{c} EditSim\left(s\left(i\right),s\left(\hat{j}\right)\right)=1-\frac{edit(s\left(i\right),s\left(\hat{j}\right))}{max(len\left(s\left(i\right)\right),len\left(s\left(\hat{j}\right)\right))}, \end{array}$$ (3)
  where *len*(⋅) represents the length (i.e., total number of characters) of the SMILES, and *s*(*i*) and $s(\hat{j}))$ are the smiles representation for drugs *i* and *j* respectively.
- Normalized longest common subsequence (NLCS) [24, 26]—The longest common subsequence (LCS) algorithm aims to find the longest common subsequence of characters between two strings. In our case, we use it to detect subsequences of characters shared by two SMILES *s*(*i*) and $s(\hat{j})$. We denote LCS of *s*(*i*) and $s(\hat{j}))$ by $LCS(s\left(i\right),s\left(\hat{j}\right))$. The NLCS is computed by normalizing LCS with the following formula:
  $$\begin{array}{c} NLCS\left(s\left(i\right),s\left(\hat{j}\right)\right)=\frac{len{\left(LCS\left(s\left(i\right),s\left(\hat{j}\right)\right)\right)}^{2}}{len\left(s\left(i\right)\right)\times len\left(s\left(\hat{j}\right)\right)}. \end{array}$$ (4)
- Term frequency (TF)—We represent SMILES *s*(*i*) with a vector composed of the frequency of each character *c*_*x*_ and refer to it as *TF*(*s*(*i*)) Then, we use the *cosine* similarity measure:
  $$\begin{array}{c} Cosine\left(s\left(i\right),s\left(\hat{j}\right)\right)=\frac{TF\left(s\left(i\right)\right)TF\left(s\left(\hat{j}\right)\right)}{\|TF\left(s\left(i\right)\right)\|\;\|TF\left(s\left(\hat{j}\right)\right)\|}. \end{array}$$ (5)
- Ensemble—We define an ensemble measure as the average of the predictions made with each of the similarities mentioned above measures.

Unless we specify otherwise, when utilizing the LAMFP algorithm, the ensemble serves as the default similarity measure.

## 3 Evaluation

### 3.1 Baselines

#### 3.1.1 One-Hot Encoding and GRU

We use SMILES to represent and recover the chemical structure for each drug. In SMILES, chemical atoms and bonds are denoted by characters. This method uses one-hot encoding and a gated recurrent unit (GRU) with the SMILES representation. We represent each character in each SMILES representation with a one-hot encoding vector where each vector’s size equals the number of unique characters in the dataset. Then, based on the one-hot encoding vectors, we utilize a GRU [27], which can process time-series information, capturing hidden patterns for different prediction tasks. The use of a GRU is based on the assumption that patterns in the order of characters in the SMILES representation can be used to classify a drug, as demonstrated by [28].

Using a GRU based on *ts* consecutive characters’ one-hot vectors, represented with *cl*, allows us to capture hidden relations between different drugs’ SMILES characters and leverage these connections to predict interactions between drugs. We use the same GRU for both drugs’ SMILES and concatenate the hidden representation for the drugs’ SMILES. Based on the concatenation of the SMILES hidden representation (i.e., the output of the GRU), we add a layer with a single unit and the *sigmoid* activation function to predict the DDIs.

#### 3.1.2 Char2Vec and GRU

We use Char2Vec to represent characters in the SMILES represented with a latent vector. This method is motivated by the success of word2vec, suggested by [29], to represent words in a latent space. Unlike one-hot encoding, using Char2Vec enables us to represent each character with respect to its context (i.e., the surrounding characters), capturing different patterns in the chemical structure of various drugs. In this method, we utilize the GRU based on the SMILES characters’ representations derived from Char2Vec, similar to the manner described in Section 3.1.1.

#### 3.1.3 CASTER

To compare the abovementioned methods to existing work, we utilize a framework recently introduced by [6], *CASTER*, as a baseline. The CASTER framework uses functional representations to represent the different drugs, i.e., the authors used the most frequent substructures shared by a pair of drugs. Then, the authors used an unsupervised encoder-decoder network to create a latent representation for each drug’s functional representation.

The authors also represented the most frequent SMILES substructures with a designated latent representation. Latent vectors of the functional representation are mapped to the same latent space of the SMILES substructures. Linear coefficients are used as features to predict the DDIs. In the training phase, the authors minimized two loss functions: (1) reconstruction loss, to represent the drugs’ latent functional representation, and (2) prediction loss, which is a binary cross-entropy loss function, to provide the prediction of DDIs.

#### 3.1.4 Directed message passing neural network

According to [30], using SMILES representation with a recurrent neural network is not optimal. The SMILES sequence represents the atoms of a 3-dimensional molecule in an orderly manner; additionally, ring numbers represent neighbouring atoms that were separated to allow a simplified representation. For these reasons, SMILES is not the optimal representation for accurately determining the properties of a molecule. Therefore, the authors presenting Chemprop, a message passing neural network for molecular property prediction, suggest recovering the actual chemical structure graph from the SMILES representation and processing it with a message passing neural network (MPNN). Based on this insight, and following Chemprop’s success in discovering a new antibiotic [23], we propose generating DDI predictions based on the molecules’ chemical structure by utilizing a graph convolutional model [19] and refer to it as *Chemprop* in this paper.

The MPNN framework consists of two phases: (1) the *message passing phase*, in which a latent representation represents the molecule; this phase runs in several iterations to update the bonds’ and atoms’ latent representation; (2) the *readout phase*, in which a readout function is used to compute the prediction using the representation of the whole graph. Using the MPNN framework can result in a noisy graph, and a less accurate representation due to totters [31]. Therefore, the Chemprop framework employs a directed MPNN (D-MPNN) in which the messages are associated with directed edges (bonds) instead of the atoms.

#### 3.1.5 SSI-DDI

SSI-DDI [7] is a recently released DDI prediction system that extracts features from raw molecular graph representations of pharmaceuticals. The model is based mainly on several graph attention (GAT) layers followed by a co-attention layer. SSI-DDI is trained to distinguish between different drug interaction types; the algorithm samples negative instances from the training set. Here, to adapt SSI-DDI to the current problem, we train SSI-DDI on just a single target attribute because the current work defines the task as binary.

We implement all the methods presented above in Python using the TensorFlow library and the default hyperparameters for CASTER, SSI-DDI, AMFP, and Chemprop. For the Char2Vec model, we use an embedding size of 100. For LAMFP, we set the *m* value to three, which is the average number of similar drugs for drugs in DrugBank.

### 3.2 Dataset

To evaluate the proposed methods, we use two versions of DrugBank [32]; version 5.1.3, released in April 2019, is used as the training set, and version 5.1.6, released in April 2020, is used as the test set. In DrugBank 5.1.6, we identify new drugs that were not part of version 5.1.3, i.e.; we removed drug pairs that appeared in version 5.1.3. DrugBank also provides information regarding each drug’s chemical structure found using the drug’s SMILES representation. Only drugs with SMILES representation available were used in this research. The number of drugs and interactions for the two versions of DrugBank is presented in Table 1.

**Table 1:** DrugBank’s drug-drug interaction statistics (only new information is presented for version 5.1.6). The two releases were used to perform a retrospective analysis.

Version	5.1.3	5.1.6
Number of drugs	3,478	843
Positive interactions	1,237,987	221,449
Negative interactions	5,383,863	2,490,061

### 3.3 Experiment setup

#### 3.3.1 Preprocessing

We only use drugs with at least one interaction with another drug. Additionally, we removed drugs with no SMILES representation. The final train and test sets consist of 2,847 and 530 drugs, respectively.

#### 3.3.2 Validation

We evaluate the methods on the following test subsets:

- Known-New Interactions—We use all drug pairs (i.e., positive and negative cases, or, existing and non existing drug interactions), where each pair is composed of an existing drug from DrugBank 5.1.3 and a new drug added to DrugBank 5.1.6.
- New-New Interactions—We use all drug pairs among just the new drugs found in DrugBank 5.1.6.
- All Interactions—We use all possible drug pairs (i.e., positive and negative cases, or, existing and non existing drug interactions). This subset is equal to the complete test set defined above.

**Fig 3:**
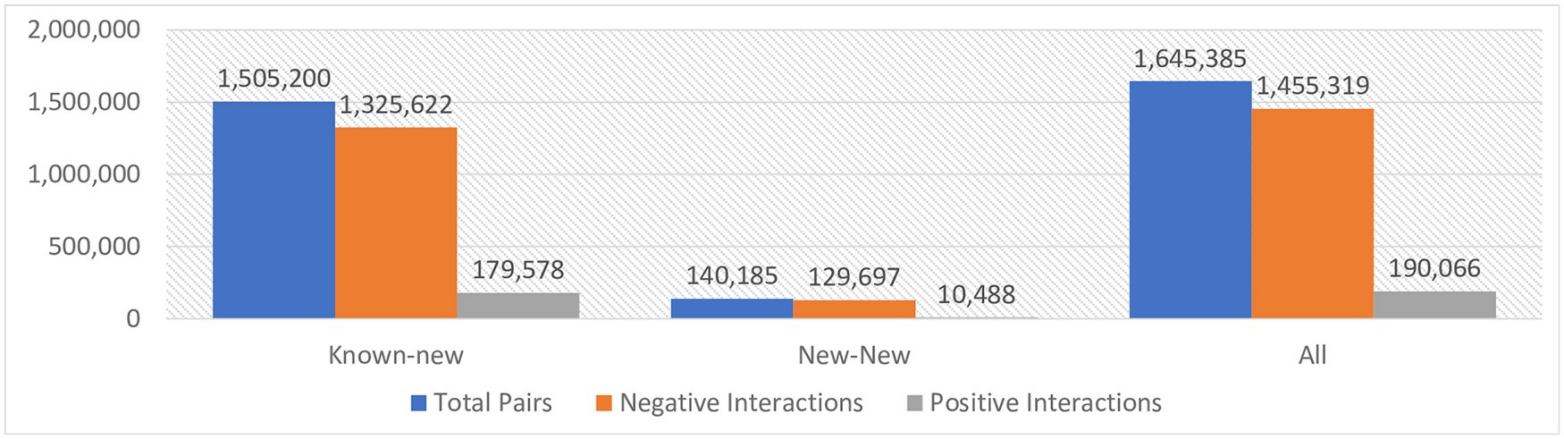
Comparative distribution of negative and positive drug-drug interactions across three test subsets. This bar chart displays the distribution of negative (non-existing according to current knowledge) and positive (existing) drug-drug interactions across three distinct test subsets: the comprehensive subset (denoted as ‘All’), the subset with new-new drug pairings, and the subset with known-new drug pairings. It’s evident that negative interactions prevail in each subset, while positive interactions constitute a considerably smaller segment of the analyzed pairs.

The distribution of positive and negative interactions within these subsets is visually depicted in Fig 3. It is worth noting that DrugBank provides data only on interactions that are known to exist. Interactions that do not appear in the database can be classified as unknown; some truly do not exist, while others might not have been discovered yet. For the purpose of both training and evaluating our models in this manuscript, we operate under the assumption that all non-existing interactions genuinely do not exist. The primary objective when ranking these interactions is to identify those that are currently unknown but do indeed exist. Adopting this methodology aligns with common practices in various computer science domains, notably in recommender systems and link prediction.

#### 3.3.3 Evaluation metrics

The following metrics are used to evaluate the proposed methods:

- AUC—The area under the ROC curve (AUC) reflects the average performance of the classifier with different classification thresholds. Used as the main evaluation metric.
- Mean reciprocal rank (MRR)—We measure the mean reciprocal rank of the first existing interaction in the ordered list of predictions. We report this metric based on the assumption that identifying the first correct interaction is highly important.
- Mean average precision (MAP)—The MAP is calculated based on the mean of the average precision for each drug.
- Area under the precision-recall curve (AUPR)—which is appropriate for rare events and is not dependent on model specificity.

For all metrics, higher values indicate better performance.

To investigate the role of molecular similarity and weight in prediction accuracy, we analyzed their correlation with prediction errors. This analysis aimed to discern the efficacy of current similarity metrics, especially when dealing with heavier molecules.

### 3.4 Results

Table 2 presents the MRR and AUC for all methods for all test subsets. The table shows that for all subsets, the highest AUC and MRR were achieved by the LAMFP method. This result demonstrates the benefit of using the lookup mechanism to predict DDIs. We performed a Friedman test on the AUC values presented in Table 2 and report *P* < 0.002. We also compared the best performing method, LAMFP, to all other methods using the analysis suggested by [33] and report *P* < 1 × 10^−5^ for all comparisons. Chemprop’s performance was the second-best; this result demonstrates the capabilities of a message-passing neural network in the current task. CASTER outperformed the Char2Vec, one-hot encoding, and SSI-DDI models, which implies an advantage of CASTER from generating functional representations using substructures information from the SMILES representation; however, the SMILES-based methods (including CASTER) underperformed in the current task. AMFP’s results were relatively low; this result can be explained by the model’s inability to deal correctly with unseen drugs, which are the samples our experiments focus on. As part of the experiments, we also created an ensemble of the different methods (see Table 2). However, it did not improve the results. We assume that this result is the high difference between the best and worst-performing methods.

**Table 2:** The AUC, MRR and AUPR values for all methods and test subsets. (Bold: Best score).

Test subset	All			Known-New			New-New		
Measure	AUC	MRR	AUPR	AUC	MRR	AUPR	AUC	MRR	AUPR
Char2Vec	0.724	0.399	0.228	0.726	0.407	0.235	0.691	0.226	0.141
One-Hot	0.707	0.372	0.216	0.709	0.372	0.223	0.669	0.165	0.132
CASTER	0.741	0.418	0.252	0.745	0.416	0.259	0.648	0.295	0.131
SSI-DDI	0.666	0.280	0.200	0.667	0.332	0.208	0.647	0.207	0.122
Chemprop	0.780	0.425	0.293	0.785	0.494	0.304	0.705	0.307	0.160
AMFP	0.690	0.510	0.218	0.704	0.510	0.227	0.466	0.000	0.069
LAMFP	**0.815**	**0.541**	**0.338**	**0.819**	**0.541**	**0.346**	**0.744**	**0.310**	**0.206**

We additionally showcase the outcomes concerning accuracy, specificity, and sensitivity in Table 3. It is evident that although the LAMFP did not attain the highest performance in specificity and sensitivity individually, it demonstrated superior results when considering a balanced combination of both metrics.

**Table 3:** The accuracy, specificity, and sensitivity measurements for all methods and test subsets. (Bold: Best score).

Test subset	All			Known-New			New-New		
Measure	Accuracy	Specificity	Sensitivity	Accuracy	Specificity	Sensitivity	Accuracy	Specificity	Sensitivity
Char2Vec	0.7669	0.8078	0.4545	0.7667	0.8088	0.4564	0.7698	0.7979	0.4227
One-Hot	0.7986	0.8592	0.3353	0.7963	0.8583	0.3385	0.8242	0.8681	0.2810
CASTER	0.8131	**0.8655**	0.4118	0.8088	**0.8610**	0.4237	0.8596	0.9122	0.2092
SSI-DDI	0.6798	0.6987	0.5356	0.6802	0.6997	0.5365	0.6757	0.6882	**0.5201**
Chemprop	0.7894	0.8170	**0.5781**	**0.8797**	0.8174	**0.5848**	0.7867	0.8128	0.4642
AMFP	0.7707	0.8170	0.4167	0.7564	0.7991	0.4410	**0.9252**	**1.0000**	0.0000
LAMFP	**0.8249**	0.8614	0.5457	0.8216	0.8574	0.5574	0.8608	0.9024	0.3462

**Fig 4:**
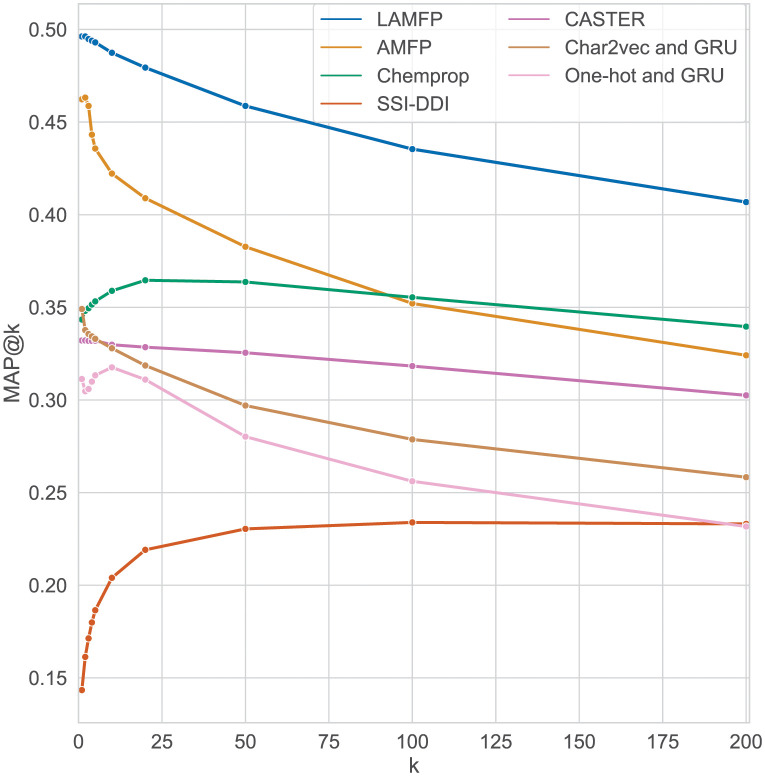
MAP@k for all methods and test subsets.

Fig 4 presents the MAP@k for all methods for different *k* values and the full test set (all interactions). As seen in the figure, for all *k* values, LAFMP obtained the best results. For *k* values under 100, AFMP achieved the second-best results; for *k* values over 100, Chemprop obtained the second-best results.

The AUC and MRR for LAMFP for all similarity measures and test subsets are presented in Table 4. Interestingly, the best results in terms of AUC were achieved by an ensemble combining all of the similarity measures. This could indicate that combining two types of similarity measures (i.e., the similarity between molecules’ chemical representation measures and SMILES-based measures) provides superior results. We additionally showcase the outcomes for accuracy, specificity, and sensitivity in Table 5 for different similarity measurements. We can observe that the ensemble-based approach demonstrated superior performance in terms of accuracy and specificity measurements. In contrast, concerning sensitivity measurement, both the Tanimoto and ED methods outperformed the ensemble. This discrepancy suggests that the ensemble’s strength lies in optimizing accuracy and specificity, while the Tanimoto and ED methods excel in enhancing sensitivity.

**Table 4:** The AUC, MRR and AUPR measurements for all similarity measures with LAMFP for all test subsets. (Bold: Best score).

Test subset	All			Known-New			New-New		
Measure	AUC	MRR	AUPR	AUC	MRR	AUPR	AUC	MRR	AUPR
Tanimoto	0.809	0.525	0.338	0.814	0.53	**0.348**	0.733	**0.310**	0.191
ED	0.783	0.525	0.304	0.788	0.526	0.313	0.736	0.279	0.171
NLCS	0.761	0.496	0.293	0.768	0.502	0.304	0.641	0.244	0.144
TF	0.760	0.525	0.270	0.767	0.525	0.280	0.647	0.207	0.130
Ensemble	**0.815**	**0.541**	**0.338**	**0.819**	**0.541**	0.346	**0.740**	**0.310**	**0.206**

**Table 5:** The accuracy, specificity, and sensitivity measurements for all similarity measures with LAMFP for all test subsets. (Bold: Best score).

Test subset	All			Known-New			New-New		
Measure	Accuracy	Specificity	Sensitivity	Accuracy	Specificity	Sensitivity	Accuracy	Specificity	Sensitivity
Tanimoto	0.8047	0.8278	**0.6277**	0.8060	0.8293	**0.6343**	0.7903	0.8126	**0.5150**
ED	0.7961	0.8264	0.5640	0.7963	0.8267	0.5714	0.7946	0.8236	0.4363
NLCS	0.8081	0.8521	0.4707	0.8062	0.8503	0.4807	0.8280	0.8708	0.2985
TF	0.7848	0.8216	0.5032	0.7845	0.8214	0.5124	0.7878	0.8235	0.3457
Ensemble	**0.8249**	**0.8614**	0.5457	**0.8216**	**0.8574**	0.5574	**0.8608**	**0.9024**	0.3462

### 3.5 Influence of molecular similarity and weight on prediction accuracy

**Fig 5:**
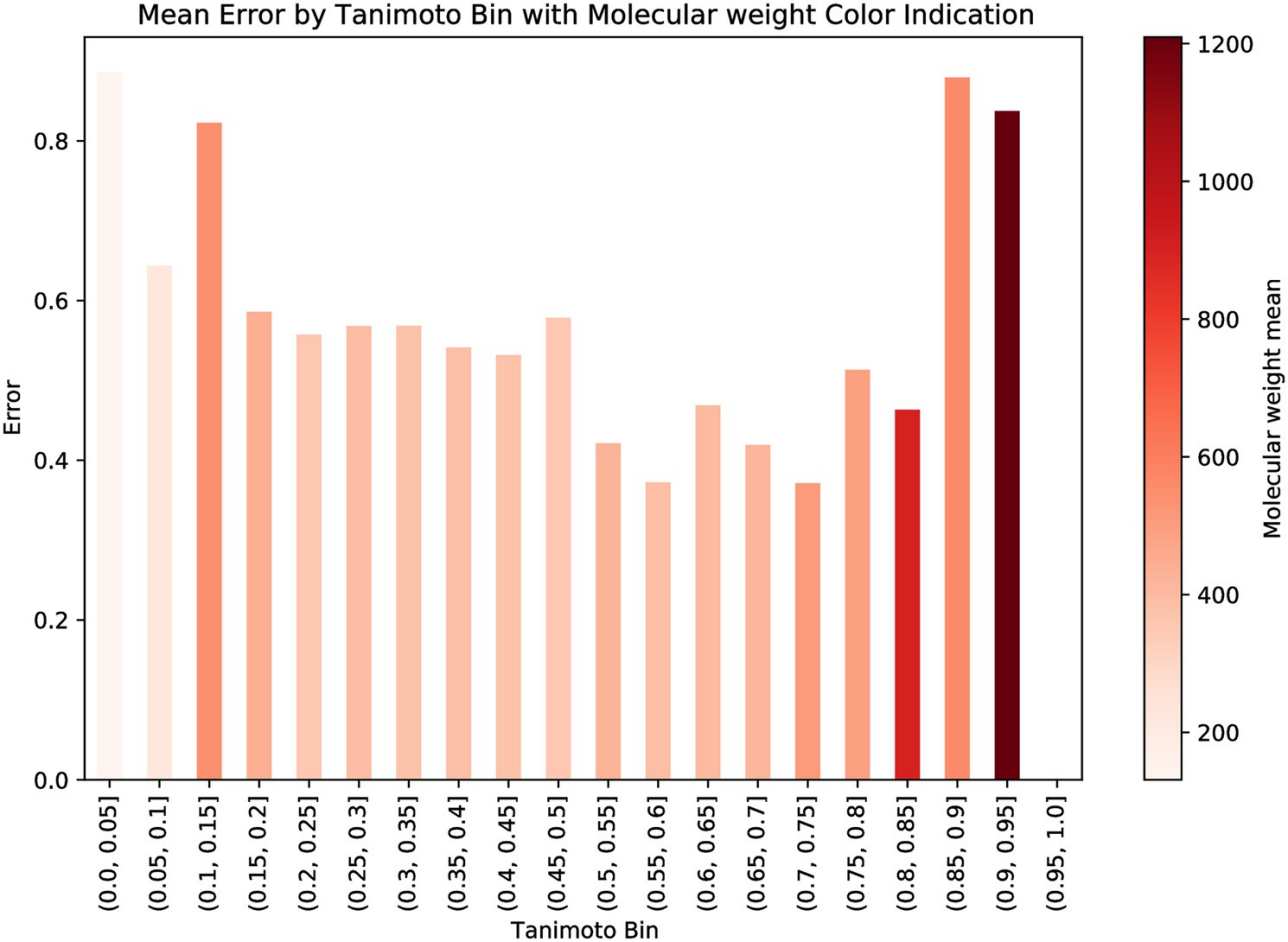
Plot illustrating prediction error against molecular similarity, with color gradient denoting molecular weight. Heavier molecules tend to show lower prediction accuracy.

In evaluating the applicability domain of our model, we examined the relationship between molecular similarity and prediction error. Our results, as represented in Fig 5, show a clear inverse correlation between molecular similarity and prediction error, indicating that as molecules become more similar, the prediction accuracy of our model increases. Interestingly, when taking into account molecular weight (as indicated by the color of the bars), a distinct pattern emerges. Heavier molecules, despite their similarity, generally exhibit lower prediction accuracy. This suggests that while molecular similarity plays a key role in enhancing prediction precision, molecular weight introduces an additional layer of complexity. This underscores the importance of considering molecular weight as a potential factor influencing the reliability of our model’s predictions, especially for heavier molecules.

In trying to elucidate the observed discrepancy in prediction accuracy for heavier molecules, several explanations surface. First and foremost, the inherent complexity of heavier molecules likely leads to a more intricate drug-drug interaction (DDI) fingerprint. With their augmented structural complexity, these molecules frequently possess a broader spectrum of potential interaction sites, magnifying the difficulties in accurately capturing and forecasting their interactions. Secondly, there may be inherent limitations in the molecular similarity metrics we’ve employed, particularly when dealing with the subtleties of larger molecules. While these metrics effectively delineate similarities among smaller molecules, they may not translate as adeptly to the nuances of their heavier counterparts. Moreover, there’s the possibility of biases in our training data potentially influencing the outcome, especially if there was an under representation or lack of diversity of heavier molecules in the dataset. These factors, either in isolation or synergistically, might be at the helm of the observed predictive variations. Exploring alternative metrics or methods specifically tailored for tackling large molecules remains an avenue for future work.

## 4 Discussion

This paper tackled the preclinical, cold-start DDI prediction problem using a simple yet effective chemical similarity-based method. We introduced a new method, LAMFP, which extends AMFP [11] by adding a lookup mechanism that uses information about similar existing drugs to predict the DDIs of new drugs. We also compared various DDI prediction methods based on different principles and demonstrated the superiority of our simple, straightforward similarity-based method over complex state-of-the-art models. The proposed lookup mechanism is motivated by TTA, a technique that has been used successfully in other domains [21, 22]. LAMFP uses existing drugs as augmentations of new drugs, which was shown to be a simple and efficient method for DDI prediction in new drugs.

The originality of this work lies in the fact that we: (1) mathematically define the preclinical DDI prediction task; (2) propose a lookup mechanism for the preclinical prediction of DDIs; (3) evaluate the impact of different types of similarity measures: chemical-structure and SMILES-based measures; and (4) experimentally evaluate multiple preclinical DDI prediction algorithms. These contributions allowed us to create an accurate model for DDI prediction in new drugs that can be used at an earlier stage in drug development than existing methods. This has the potential to save lives and potential costs if, for example, there are critical negative interactions between a new drug and many commonly used drugs.

To demonstrate the applicability of our method, we explore the top ten correct positive known-new DDIs predictions detected by employing our model with our test set. We highlight the fact that with the known-new settings, our method did not train over the new drugs, thus, we simulate real settings to discover interactions between known and new drugs. The results revealed interesting interactions, such as the drugs Clothiapine and Imipramine. The combination of these drugs is particularly interesting due to the distinct pharmacological profiles of the two drugs and the potential implications for DDIs. Clothiapine is an atypical antipsychotic medication primarily used to treat schizophrenia and other psychotic disorders, while Imipramine is a tricyclic antidepressant often prescribed for the treatment of depression and various anxiety disorders. Although these drugs have dissimilar mechanisms of action, co-administration of these drugs might increase the risk of adverse effects (https://go.drugbank.com/drugs/DB00458).

Among the top ten correct positive predictions on the test set we also found another intriguing drug pair, Terguride and Risperidone. The combination of Terguride and Risperidone poses a potential risk for the severity of hypertension (https://go.drugbank.com/drugs/DB13399). Terguride has been explored for Parkinson’s disease and migraine treatment, while Risperidone is an antipsychotic agent used for schizophrenia and bipolar disorder. The complexity of these conditions and the varying mechanisms of action of the drugs increase the likelihood of unwanted interactions, underscoring the need for caution. In both cases, the potential for harmful interactions highlights the significance of predictive models in identifying potential risks, thereby enabling healthcare professionals to make informed decisions and mitigate potential adverse outcomes when prescribing multiple medications to patients.

Additionally, we perform error analysis, unveiling instances where drugs were erroneously classified as positive interactions despite the absence of any actual interaction. Notably, in six out of the ten cases where our model assigned high probabilities to positive interactions that did not materialize, the drug Nifedipine emerged. Nifedipine, a calcium channel blocker harnessed for treating hypertension and angina, appears in these instances. The model’s tendency to mistakenly predict interactions might stem from shared molecular attributes, resulting in false positives. This hints at the model’s propensity to associate Nifedipine with other drugs due to structural or pharmacological similarities, leading to inaccurate predictions. Given Nifedipine’s widespread usage and multifaceted pharmacological effects, it might function as a “hub” drug in our model, establishing connections with various other medications.

We evaluated the proposed method by performing a retrospective evaluation using two versions of the DrugBank database. Our robust evaluation focused on new drugs added to a later version of the database. We compared the proposed methods to existing state-of-the-art methods and reported the AUC, MRR, AUPR, and MAP@k. Focusing on the full test set, we can see that the LAMFP algorithm performed best, outperforming the second-best method, Chemprop, by 4% in terms of the AUC and 12.6% in terms of the MRR. For the other interaction types (e.g., known-new and new-new interactions), LAMFP was the only method that showed consistently strong performance. These results suggest that simple, similarity-based methods are preferred over complex models and should be used as a baseline when evaluating new methods. LAFMP’s outstanding performance compared to the other advanced methods examined indicates that the simple lookup mechanism proposed is an essential part of our method.

We explored the use of various similarity measures in the lookup mechanism and showed that an ensemble of different similarity scores performed best. In most cases, the best performing single similarity measure was based on calculating the Tanimoto coefficient on the Morgan fingerprints of the molecules. We additionally showcase the outcomes of accuracy, specificity, and sensitivity across all similarity measurements. The findings revealed that the ensemble method exhibited the highest level of accuracy and achieved superior outcomes in terms of specificity. These outcomes underscore the ensemble’s effectiveness in accurately identifying positive drug interactions, thereby minimizing false positives. Nevertheless, in terms of sensitivity, the ensemble method fell short in comparison to the Tanimoto and ED approaches. This discrepancy could imply that the ensemble method prioritizes the reduction of false positives, potentially resulting in a compromise on sensitivity. This trade-off highlights the intricate balance between minimizing false positives and maximizing true positives in the context of drug interaction prediction. Like the NLCS and ED, the TF measure is calculated by processing the SMILES representation directly and not the actual chemical structure; in most cases, their use resulted in poorer performance than that of the similarity measure that processes the actual chemical structure. Evaluating different similarity measurements that capture different features (e.g., a string-based feature when working with SMILES) may be useful in future work on predicting the drug properties of new drugs.

In our methodology, we employ the SMILES representation for most of the similarity measures in LAMFP, excluding the Tanimoto similarity based on fingerprints. It’s pertinent to mention that we do not utilize canonical SMILES in this study. Previous research by Bjerrum [34] has indicated the potential for improved performance when using multiple SMILES due to the augmentation of samples and the addition of more diverse data to the model. Different SMILES representations for the same molecule can provide varying data perspectives, enriching the model’s learning. Though we recognize the possible advantages of canonical SMILES, we have chosen our current approach for the present study. We believe that further exploration of canonical SMILES and its subsequent influence on the model is a valuable avenue for future work.

Delving deeper into the role of molecular similarity in DDI prediction, we analyzed its correlation with prediction error while considering molecular weight. This analysis revealed that as molecular similarity increased, prediction error generally decreased. However, a notable variance was observed in heavier molecules, which consistently exhibited lower prediction accuracy. Two plausible explanations underpin these findings: firstly, heavier molecules tend to have more intricate drug-drug interaction fingerprints, complicating DDI predictions. Secondly, our current similarity metrics may not be as effective when grappling with heavier molecules, hinting at the necessity for alternative or supplementary metrics to address these molecules. Exploring other metrics tailored to handle larger molecules will be an avenue for future research.

Previous studies showed that known DDIs of drugs are of high importance for predicting new DDIs [11], and our results suggest that even if no existing DDIs have been discovered for a new drug, the DDIs of similar known drugs can be used for DDI prediction by utilizing a similarity measure. This is reflected in the difference between the performance of the LAMFP and AMFP methods. AMFP was not designed to handle new drugs; therefore, it performs poorly on new drug interactions. Unlike AMFP, LAMFP calculates a new drug’s prediction using the predictions for drugs that were part of the training set, based on a similarity measure. AMFP’s relatively high MRR value of 0.5513 on the known-new test subset demonstrates that at least one interaction for a new drug can be predicted without any information regarding that drug, relying only on the interaction information about the other, existing drug. This might be explained by the principle of popularity, where some drugs tend to interact more than others.

Our results also demonstrate the capabilities of a message passing neural network (Chemprop) in the task of DDI prediction for new drugs. Furthermore, our results support the claim that using the chemical structure to represent a drug is preferred over training a recurrent neural network with SMILES representations, as Chemprop outperformed all of the SMILES-based methods (i.e., Char2Vec, one-hot encoding, and CASTER). Chemprop uses the chemical structure graph recovered from the SMILES representation and processes it with an MPNN, a special graph neural network type. The model based on Char2Vec outperformed the one-hot encoding model as expected; this result demonstrates the contribution of using character embeddings over the simple one-hot encoding representation. Lastly, we note that CASTER’s results were lower than reported in the original paper presenting CASTER; this difference can be attributed to the sampling performed on the test set in the original work, which was not performed in our study. The results of all examined baselines indicate that some algorithms that were not aimed at predicting the DDIs of unseen drugs can still be effective for this task with high accuracy (e.g., Chemprop).

The major limitation of this work is our formulation of the problem as a binary problem, which does not consider the complex nature of DDIs, as there are different types of DDIs, and the severity of DDIs can vary. Another limitation stems from the fact that we only evaluated our method on a single database, DrugBank (the main database used for DDI prediction), which consists of a homogeneous set of drugs, however, this limitation is offset by the fact that we performed a retrospective evaluation on it. We also note that LAMFP, like the other methods presented in the paper, does not support biologics. Still, unlike all other methods presented here, it can be extended for that case by using a suitable similarity measure, such as a proper sequence alignment algorithm.

Our proposed method for preclinical DDI prediction can improve the drug development process and, more specifically, can assist in the identification of candidate molecules with low chances of major drug interactions while the drug is still being developed rather than when a drug is brought to market. Several drugs have been withdrawn from the market due to drug interactions (e.g., Iproniazid, https://go.drugbank.com/drugs/DB04818) Mibefradil [35], and Sorivudine [36]), and our proposed method could help reduce such incidents. Furthermore, the proposed method can be used to solve other drug-related tasks, such as predicting drug side effects and synergistic drug pairs. Our lookup mechanism supports unseen drugs and can generalize these problems and other drug property prediction models. Examples include drug pregnancy safety prediction [37], where compound structure can be used by the lookup mechanism to identify similar drugs. Another such example is lactation safety prediction. In future work, we plan to extend the use of our model, ensuring that it supports biologics by defining a suitable distance measure and evaluating the performance of this type of drug.
